# Look out for Small Pox

**Published:** 1882-12

**Authors:** 


### LOOK OUT FOR SMALLPOX.

The warning comes from the South ; smallpox is prevalent in Chattanooga, Tenn. ; so also is it, nearer home. A dispatch from Havre-de-Grace, Md., tells us that Harford county is much excited over the prevalence of smallpox, which was introduced into that community last summer by some men coming to work in a canning factory.

You must ever bear in mind that the causative agents of smallpox can be carried great distances and produce the disease. A man may come from a region in which the disease is prevailing, and while perfectly well himself, may carry in his clothing, his beard or his hair, the seeds of smallpox, thus constituting a nucleus from which may originate a most devastating epidemic. The following fact must ever be borne in mind : *He who comes at all into contact with strangers is exposed to a greater or lesser degree of danger of infection.* The remedy, or rather the prevention, is to be found in thorough and universal vaccination and re-vaccination.—*Med. and Surg. Rep.*

